# Learners’ Perception of Scientific Text Layouts Design Using Eye-Tracking

**DOI:** 10.3390/jemr18030022

**Published:** 2025-06-13

**Authors:** Elizabeth Wianto, Hapnes Toba, Maya Malinda

**Affiliations:** 1 Faculty of Humanities and Creative Industries, Universitas Kristen Maranatha, Bandung 40164, Indonesia; 2 Faculty of Smart Technology and Engineering, Universitas Kristen Maranatha, Bandung 40164, Indonesia; hapnestoba@it.maranatha.edu; 3 Faculty of Digital Business and Law, Universitas Kristen Maranatha, Bandung 40164, Indonesia; maya.malinda@eco.maranatha.edu

**Keywords:** adult learner, area of interest, eye-tracking, information processing, lifelong learning

## Abstract

Lifelong learning, particularly in adult education, has gained considerable attention due to rapid lifestyle changes, including pandemic-induced lockdowns. This research targets adult learners returning to higher education after gap years, emphasizing their preference for technology with clear, practical benefits. However, many still need help operating digital media. This research aims to identify best practices for sustainably providing digital scientific materials to students by examining respondents’ tendencies in viewing journal article pages and scientific posters, with a focus on layout designs that include both textual and schematic elements. The research questions focus on (1) identifying the characteristics of Areas of Interest (AoI) that effectively attract learners’ attention and (2) determining the preferred characteristics for each learner group. Around 110 respondents were selected during the experiments using web tracking technology. Utilizing this web-based eye-tracking tool, we propose eight activities to detect learners’ perceptions of text-based learning object materials. The fact that first language significantly shapes learners’ attention was confirmed by time-leap analysis and AoI distances showing they focus more on familiar elements. While adult learners exhibit deeper engagement with scientific content and sustained concentration during reading, their unique preferences toward digital learning materials result in varied focus patterns, particularly in initial interest and time spent on tasks. Thus, it is recommended that lecturers deliver digital content for adult learners in a textual format or by placing the important parts of posters in the center.

## 1. Introduction

Lifelong learning is one of the major themes in the growth of the human capital index in the global community. Since life expectancy is getting longer and the world population is getting older, attention given to the older cohort is becoming more intense. Castro states that various parties must consider four critical pillars to maintain an older adult’s well-being: health, lifelong learning, participation, and security [1].

The ability and motivation to engage in continuous educational processes, both formal and informal, significantly impact a community’s progress. As providers of general education services, universities are expected to provide various facilities to support lifelong learning inclusively for undergraduate and graduate students [2]. Especially in the post-pandemic era, individuals must keep racing to develop themselves. The presence of technological devices provides an excellent alternative for communication and access to various learning resources. In this context, our research on learners’ perceptions of scientific textual layout designs based on eye-tracking technology is timely and relevant, making the audience feel connected and involved in the current and future educational landscape.

With various material objects, online media can become a learning tool that supports lifelong learning. However, in a university, students also come from different age groups, sometimes showing significant differences in their attitudes and perceptions of learning objects. Therefore, learning objects at universities should be evaluated for their usability to serve as a guide to help students understand the material presented. The delivery of university course materials utilizes learning management tools that accommodate various types of learning content, which are then provided to students both asynchronously and synchronously. Multimedia learning, as fundamentally conceptualized by Mayer, is based on three main assumptions: (1) the dual-channel assumption (processing information through separate channels for images and words), (2) the limited-capacity assumption, and (3) the active-processing assumption, which involves integrating new information with prior knowledge [3]. Consequently, multimedia learning is more effective but can only occur when cognitive load is properly managed through appropriate multimedia design, thereby stimulating active learning engagement [4].

Adult learners of today continue to learn voluntarily or necessarily because they are encouraged to do so by their social surroundings [5]. This choice of activity is needed to maintain active citizenship in economic and cultural fields for an ever-changing society, even though it requires the balancing of multiple responsibilities and overcoming of financial aid policy constraints [6] to return to school. Additionally, it is well recognized that a person’s motivation to learn decreases with age or with fewer educational accomplishments, and, combined with that, the necessity of learning to operate devices is greater than in the last decade and even more complicated [7] for students. By raising awareness about returning to school, this older learning group needs to be helped through the transition period by using general learning media accessed by regular learners.

As acknowledged by researchers, the behaviour of adult learners who resume their studies after a gap year has not yet been linked to unconscious fixation and duration in relation to the visual layout of learning materials. Therefore, this paper addresses two research questions: (i) what characteristics of Areas of Interest (AoI) can attract learners’ attention, and (ii) which characteristics are preferable for specific learner groups? To evaluate the first question, we assessed the layout of various locations of abstracts, column arrangement, and table positions. We also examine the number and position of AoIs in a particular paper and scientific poster. To address the second question, we assessed the gaze and duration of fixation time of several textual layouts. Two kinds of respondent groups are evaluated: adult learners and regular learners.

## 2. Literature Review

Eye-tracking technology has allowed academics to investigate how different learning media might be applied in ways that go beyond more conventional approaches that rely on respondent reporting for data collection. These include improving the reading ability of students who undertake distance learning [8], increasing the use of multimedia [9], and exploring the effectiveness of new media for accessing learning materials [10]. A study utilizing eye-tracking technology has been conducted to compare the time-based visual observation processes between expert and non-expert groups, specifically regular learners, and adult learners [11]. Based on these findings, we propose to utilize web tracking technology to explore students’ gaze activities and fixation on diverse textual layout designs, as this tool can accurately capture and characterize the learning process [12], even in detecting subtle differences. Some recent research shows that web-based and mobile applications have layout structures that should be implemented to meet the user’s viewing behaviours [13].

In scientific journal and poster layout designs, we evaluated gaze perception based on fixation to predetermined objects known as areas of interest (AoI) [14] and, in the AoI, placed fixation, which refers to the eyes focusing on a specific point, such as a word within a sentence or an object in an image, although this duration may be brief, it is considered the moment during which visual information is actively processed [15].

The choice of scientific journals and posters is significant, as these two objects are highly relevant for lifelong learners who want to follow scientific developments [16], thus impacting the overall learning experience [17].

Some state-of-the-art research is directed at generating papers and posters [18,19,20]. This automatic layout design activity is mainly based on information structure and is not characterized by learners’ behaviour, especially for digital immigrants. Based on this, we hypothesize that the layout of scientific papers and posters displayed on monitors should also influence learners’ behaviours. Webcam technology devices can be used more simply for eye-tracking since eye movement can be recorded at a rate of at least 20 frames per second in an application connected to medium-speed internet [21].

Our contribution and the novelty in this research lie in the evaluation method based on the time spent on activities and AoI distances for analysing students’ gaze and fixation preferences in various scientific journals and poster designs. This research has profound practical implications, as it aims to convey good practices that support the sustainability of providing digital scientific materials to students based on acceptable design concepts and layouts. Some recent research is directed at analysing how to construct decent papers and poster layouts [18,19,20]. However, to the best of our knowledge, limited studies have explored the characteristics and behaviours of students in perceiving educational digital resources, especially for adult learners.

This study aims to determine which scientific layout has a greater impact on the learning activities of adult learners, compared to regular students. The selected course, which introduces entrepreneurship with a business plan as an output, was chosen for its relevance to the study’s focus on textual layout designs. It is an elective course in undergraduate programs and a compulsory course in the second-semester curriculum for postgraduate business management students in the selected university.

## 3. Methodology

During the experiments, an eye-tracking approach is used. It is a technological approach that can automatically quantify user behaviour to analyse adult learner interactions in real-time, evaluating visual attention based on some given interactions [22]. Eye-tracking technology developed with the primary input device as a webcam has been proven to be relatively in line with screen-based eye-trackers [23,24], and was chosen for its reliability, ease of setting up for this study [25], and the fact that it can be measured objectively compared to self-reported data collection [26].

**Figure 1:**
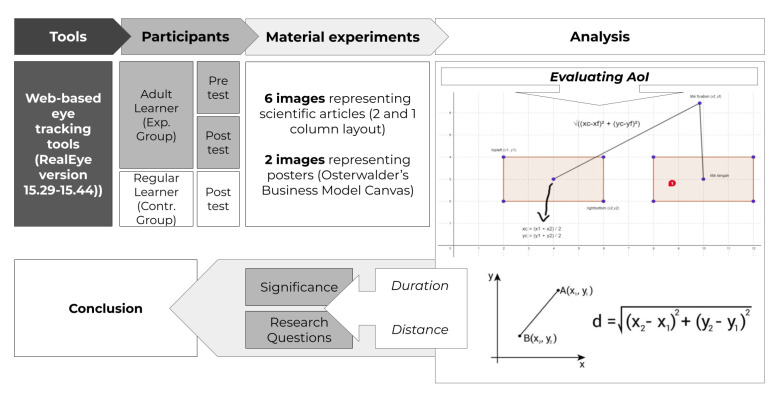
Flow of experiments.

This study was designed to incorporate both within-subject and between-subject comparisons. The results will compare the performance of adult learner participants through pre- and post-tests in a within-subject experimental design. Additionally, participants categorized as regular learners were instructed to perform a similar task but only once, serving as the control group to benchmark the scores obtained by the adult learner group. For analysis, quantitative scores were used to assess statistical significance (in Numerical Interpretation), while qualitative comparisons between the two groups were used to identify behavioural patterns of adult learner participants (in Visual Interpretation). The flow of the experiment is given in Figure 1.

### 3.1. Tools

Real Eye (version 15.29–15.44 was used for this study) is an online research platform that empowers webcams as eye-trackers. The information obtained using eye-tracking is in the form of fixation duration in certain areas, so the fixation results are considered to be the time when learners process visual information displayed in the learning management system. The quality of data collected was controlled using predefined points and background to fine-tune the model, whereby all of the respondents followed the standard calibration process containing four steps (calibration, first re-validation, large grid task, second re-validation), and, after finishing, the survey [27].

**Figure 2:**
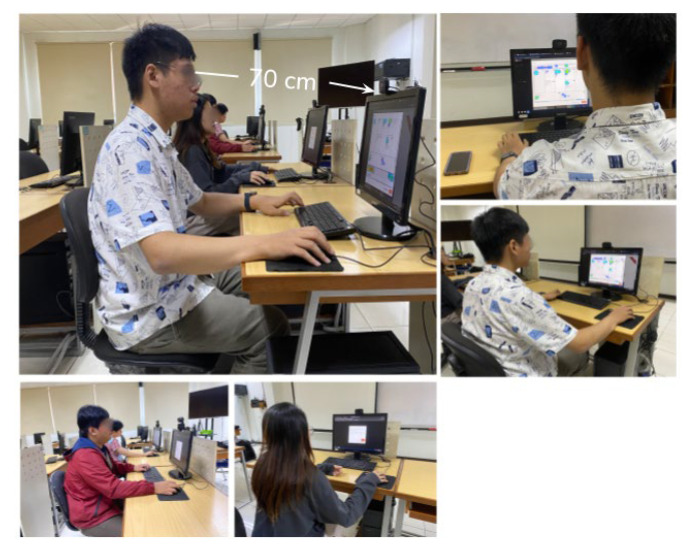
Data collection setup.

The setting for our eye-tracking environment is a 60 Hz sampling rate, 20 frames per second, which recaps gaze points every 16.6 milliseconds. A single web camera is used to facilitate interactive eye-gaze tracking. This choice is based on the minimum sampling rate for web studies [21,28]. Quantitatively, fixation duration that marks heat maps and AoIs viewed by participants will be analysed to answer research questions. The gaze and fixation data were taken for 15 s for each document layout and accessed by participants on a standard 19 (1280 × 1024 pixels) monitor in the laboratory with eye distance to web cam of approximately 70 cm. Figure 2 depicts the experimental setup used for data collection in this study.

All data collection, following the screening of prospective respondents, was recorded on the Real Eye platform at the URL: https://app.realeye.io/test/d3894aae-9f27-4ff2-87d3-c3c3c7b2b327/run (accessed on 1 March 2024) (this link is only accessible during the subscription period). The questionnaire was structured into four main sections as follows: (1) Informed consent; (2) Identity and categorization; (3) Core questions related to the scientific article; and (4) Core questions on entrepreneurship theory (business model canvas). All of the questions were conducted in the participant’s first language: Indonesian.

### 3.2. Participants

The experiment was conducted in the even semester of the 2023/2024 academic year (approximately in February to March 2024). Twenty-nine second-semester adult learners in the business graduate program participated in the experiments. They come from various backgrounds, such as medical, business, design, and engineering. We also included 83 regular students from the sixth semester of visual communication design and engineering study programs who enrolled in or have already completed the Research Methodology course.

This composition was chosen by design. Adult learners with gap years in their academic activities would have some challenges pinpointing the importance of AoIs in scientific papers and posters. In our research, an adult learner has experienced at least one gap year from high school or previous education from college [29].

However, they might already have developed the ability during their bachelor studies. For this reason, we also scheduled pre- and post-sessions to compare how adult learners perceive the textual layouts. These sessions were designed to provide a baseline and a post-experiment comparison of the learners’ perceptions of textual layouts, aiding in answering the first research question. We added the analysis of the regular students’ perceptions to answer the second research question. We only took one experiment session for this second group since they were instructed in the essential aspects of scientific paper layouts during the ‘Research Methodology’ course.

### 3.3. Material Experiments

Eight images were prepared during the experiments, including snapshots from scientific papers and posters. As Table 1 shows, the images have some specific features. The type of scientific information we explored is based on the definition of technical articles and related instructions for evaluating information structure for journals, proceedings, and posters typically formatted in A4 size (portrait, 29.7 × 21 cm) or UNESCO book size (portrait, 23 × 15.5 cm). The images used in this study are considered to represent fundamental learning materials that postgraduate students—who constitute the adult learner group in this research—are consistently advised to access and comprehend independently. The eight images referenced (as shown in Table 1) represent the following: scientific articles with single-column layouts (Images 1 and 2), scientific articles with double-column layouts (Images 3, 4, 5, and 6), and canvases representing posters or basic schematic diagrams (Images 7 and 8). Images 1 and 2 differ in the number of Areas of Interest (AoIs), highlighting the language used, similarly to Images 3 and 4, whose AoI placements differ from those in Images 1 and 2. Image 5 contains one AoI but features content showing both the abstract and main body of the article, designed with and without bolded text, distinguishing it from Images 3 and 4, which share a generally similar two-column layout. Image 6 has an AoI focused on a statistical table. Images 7 and 8 are business canvases presented as templates with concrete examples, differing in background color—Image 7 has a light background, while Image 8 has a dark one. Additionally, Image 7 includes textual information equivalent in prominence to its accompanying icons.

We presented the percentage area of the AoIs compared to the whole image. For each experiment group, we instructed the learners to find important information from the textual layout in 15 s (15,000 milliseconds) per image, the duration of each layout was based on our experience of the time required for someone to find the title, abstract, and keywords of a paper and the centrality of a scientific poster.

Before the main experiment, we asked five experienced lecturers to record the time and define their AoIs. We postulate that the area around the abstracts, such as keywords, should be the AoI of a scientific paper since important concepts are summarized in the abstracts. Since the subjects are focused on entrepreneurship, we also agreed to use the value proposition in Osterwalder’s business model canvas for the scientific posters as the central issue in defining the AoIs.

### 3.4. Analysis

To measure the importance of AoIs, we propose using vector distance computation from a gaze or fixation point to the centre of the AoI. If a textual layout has more than one AoI, we will compute several distances to each AoI centre. In our case, the vector distance is measured between two unit-length vectors in terms of pixels. Vector distances are preferable since they measure the shortest straight line distance between two points [30]. We calculate the distance to the centre point to indicate how far someone’s observation is from the centre of an AoI. We also measure the duration of each respondent in each AoI. The distance and duration would be essential to indicate how attractive the AoI is. It might be observed that someone is ‘far’ from the centre of an AoI; however, the perceived distance will be acceptable since the duration is still ‘in’ the AoI. These characteristics cannot be observed by using the usual heatmap observation. The term heatmap in this study refers to the indicated intensity of visual attention, with warmer colors or Hot Spots (red/orange) representing areas of high focus, and cooler colors or Cold Spots (blue/no color) indicating the opposite [31].

Another novelty in our study is the way in which we separate the number of seconds of each image observation into three fixation time leaps (TL) regarding duration and position, i.e., the first second, middle or median, and the last second. For instance, if a learner observes a given image for 11 s, we will take the median (the sixth second) as the middle observation time. Meanwhile, if the observation is 12 s, the middle observation will also be in the sixth second. The computation of the time leap periods [32,33] will show the general eye movement of the learners. We use the mean metrics for all observed images in all groups. The mean values will help validate the significant differences between the groups’ results using an unpaired *t*-test for two independent samples [34].

## 4. Results

We present the experimental results in several standard eye-tracking metrics, such as time to first fixation (TTFF), first fixation duration (FFD), and time spent in the AoIs (TS). TTFF measures the average amount of time in milliseconds for all respondents in a group to have a fixed look at a specific AoI. FFD measures the average time in milliseconds for all respondents in a group to fix their attention in the AoIs during the first visit, this also indicates the first visible object to grab the attention of the respondents. We argue that shorter Time to First Fixation (TTFF) values indicate faster user awareness of high-priority content. Conversely, longer First Fixation Duration (FFD) reflects prolonged engagement with critical content. In this study, ‘high-priority content’ was operationally defined using arbitrarily assigned Areas of Interest (AOIs), which varied in size and were uniformly shaped in rectangles.

TS measures the average length in milliseconds for all respondents in a group looking and concentrating on the AoIs. In this section, we present the results following the images in Table 1 and cluster them according to the group characteristics. Thus, we will have three groups: pre- and post-tests for adult learners and regular students. Our interpretations are divided into numerical, visual, and behavioural aspects.

### 4.1. Numerical Interpretation

In the numerical interpretation, we analyse the significance of each respondent group using the unpaired *t*-test for two independent samples with 95% confidence. The TTFF, FFD, and TS significance tests are computed by comparing adult learners’ pre- and post-test results. Table 2 shows the significance of the test results, which are presented in bold. All experiments’ pre- and post-test results show significant differences, with the two-tailed *p* value less than 0.0001. However, asterisks indicate the opposite of the initial expectation of the pre- and post-tests was observed.

Regarding the TTFF, we expect the duration will be shortened after some instructions. We found that the adult learners’ group significantly improved in almost all image post-test experiments, except for Image 5 and Image 8. In Image 5, the abstract of the two-columns proceeding is located in the bottom-left area, which made finding the AoI more time-consuming. In Image 8, the image is rather dark (gray-scaled), making it harder to find the intended AoI. We expect the duration of the FFD to increase in the post-test. The experiments show improvement in all images except Image 5, which relates to the TTFF results. Since it took longer to find the AoI, the learners might shorten their attention to move to the following image.

Regarding the TS, we expect the learners to stay longer in the AoI. Somehow, they intend to move more quickly to the following images during the experiment, so they generally spend less time in the AoI. Interestingly, for Image 5, where it is harder to find the AoI, the learners generally stay longer in the AoI. We assume this is because they try to ensure the AoI is located correctly.

### 4.2. Visual Interpretation

**Figure 3:**
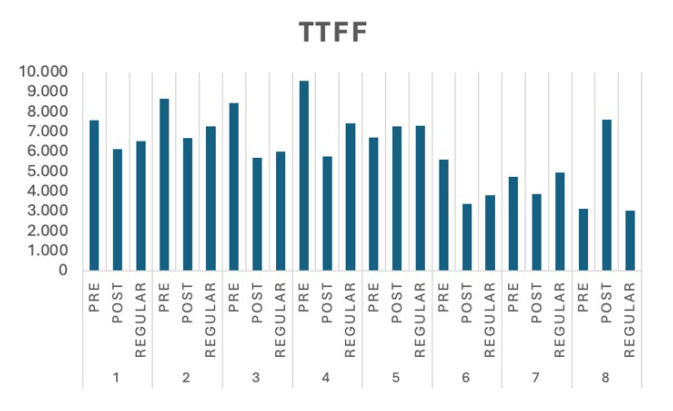
The TTFF performance of the experiment group (in ms).

**Figure 4:**
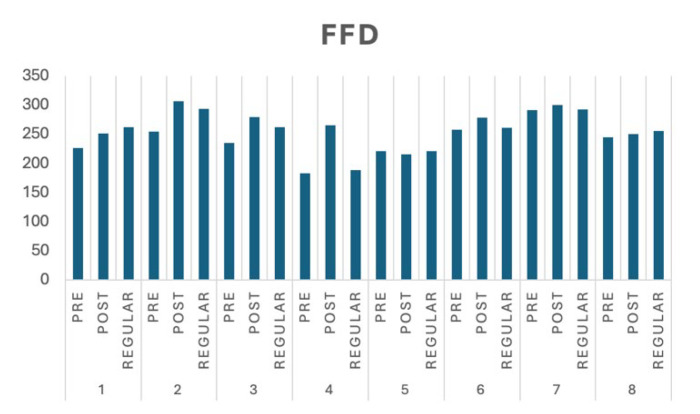
The FFD performance of the experiment groups (in ms).

**Figure 5:**
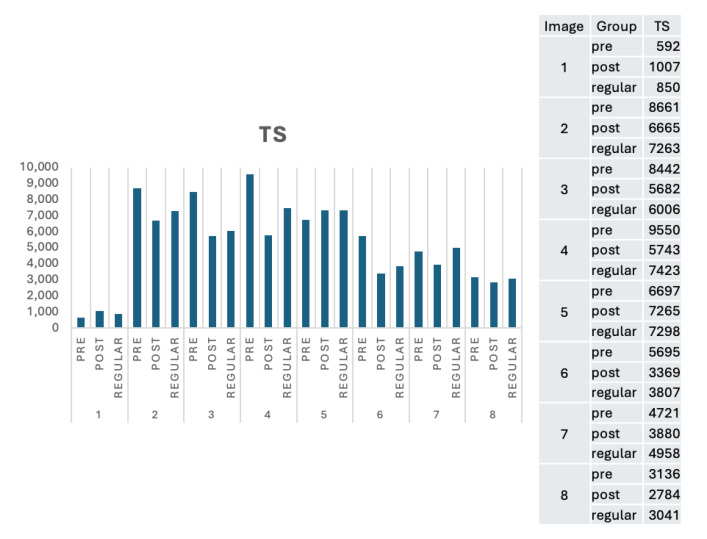
The TS performance of the experiment groups (in ms).

Figure 3, Figure 4 and Figure 5 present visualizations of the numerical results compared to a regular student. Figure 2 shows that the overall TTFF is shorter for the post-test, except for Image 8. Further observation shows that Image 8 has a dark background, and it is arguably challenging to find the exact AoI, although the learners are already familiar with the location. Regular students, in general, can find the AoIs in a relatively significantly shorter time than adult learners. This result strengthens the initial assumption that adult learners need to refresh their knowledge to be able to find the AoIs. Although regular learners take less time to find the AoIs, with more precise instructions from the lecturer, the post-test TTFT of adult learners is the same or even shorter than regular students.

In Figure 4, we can see that the FFD of the post-test for adult learners increases. This indicates that adult learners can concentrate on the AoI after further instruction during the post-test. Interestingly, Image 4 shows a significant ‘attention’ behaviour during the pre-test, which was also detected for regular learners. The side-by-side bilingual abstracts and keywords seem to shorten the period for finding the AoIs. This suggests that a single AoI with one specific language appears sufficient to show essential concepts in a scientific paper.

An interesting fact from Figure 4 can be derived from Image 1. The time the learners spent in the AoIs is significantly shorter than that of the other images. This fact suggests that once the learners find the AoIs, they directly move to the following image. A possible explanation is that the learners see the AoIs relatively quickly in one of the languages present in the paper. This fact also suggests that learners concentrate on a language they mastered. Comparable results for Image 4 are shown in Figure 4 and Figure 5. Adult learners generally pay more attention to side-by-side bilingual abstracts and keyword layouts.

### 4.3. Behavioral Interpretation

**Figure 6:**
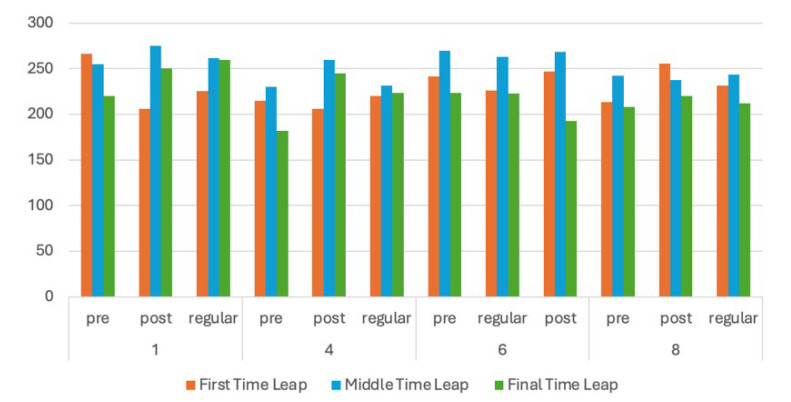
The TS performance, middle, and last duration fixation of the AoIs (in ms).

**Figure 7:**
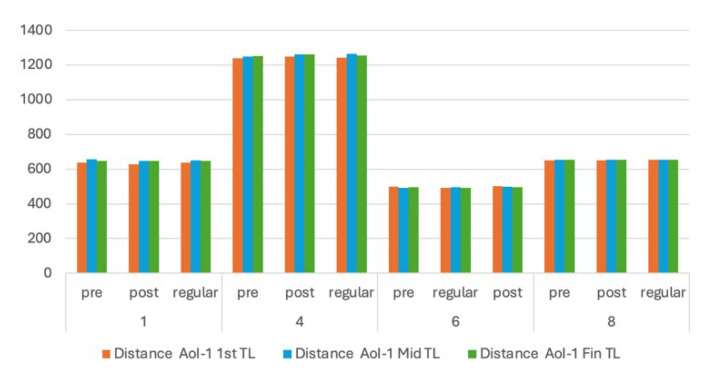
First, middle, and last distance fixation to the first AoIs (in pixels).

**Figure 8:**
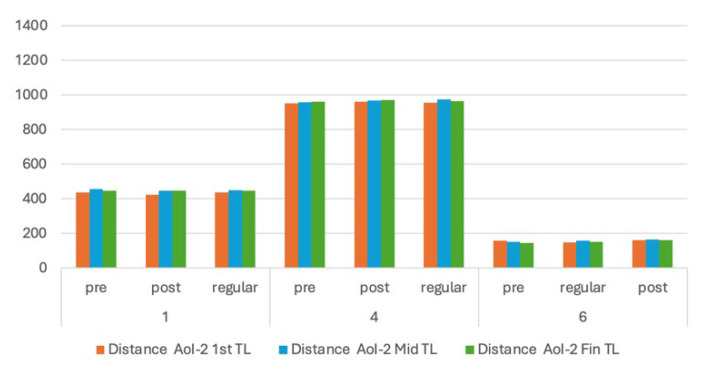
First, middle, and last distance fixation to the second AoIs (in pixels).

Based on the results, further evaluations were carried out for Images 1, 4, and 8, which showed different behaviours than the other images. We evaluated how the learners perceived the images—regarding the time spent and distance to AoIs—during the fixation time leaps in the first second, middle or median period, and the last second. We also compared those results with specific content AoIs in the form of tables in Image 6. We hypothesize that content would attract more attention from the learners. The results are presented in Figure 6, Figure 7 and Figure 8.

Figure 6 shows that the overall fixation average duration in the AoIs for all groups is around 0.2 s without significant differences in all leap periods. This suggests that during the whole experiment, all learners concentrate on the essential areas if they already know what to find. Especially in Image 6, where the important contents are presented in a paper, all groups can locate essential information from the tables. In that case, the respondents demonstrated a higher fixation duration.

From Figure 6 and Figure 7, Image 4 shows significantly higher distances in all leap periods. This fact suggests that all learners directed their attention to the side-by-side AoIs. However, we have two AoIs so that learners can concentrate on the left or right side. Furthermore, the distances of the left AoI are significantly lower than the right. This would suggest that they pay more attention to the right-side AoI given in the mother tongue. This fact also strengthens the results of Image 1, where two AoIs (the keyword) are positioned at the top and bottom and separated by another portion of text (the abstract).

For scientific posters, we believe that the learners would be able to concentrate directly on the AoI. In Figure 7, the learners showed their ability to locate their first fixations in the defined AoIs for Image 8 in a significantly shorter time, as shown in Figure 3 for the adult learners’ pre-test and the regular learners. These facts might also be correlated with the size of the AoIs. The greater area of AoIs, such as in Image 1 and Image 8, might help the learners locate their fixation faster and ensure that they follow the content.

## 5. Discussion

### 5.1. RQ1: What Characteristics of AoI Can Attract Learners’ Attention?

**Figure 9:**
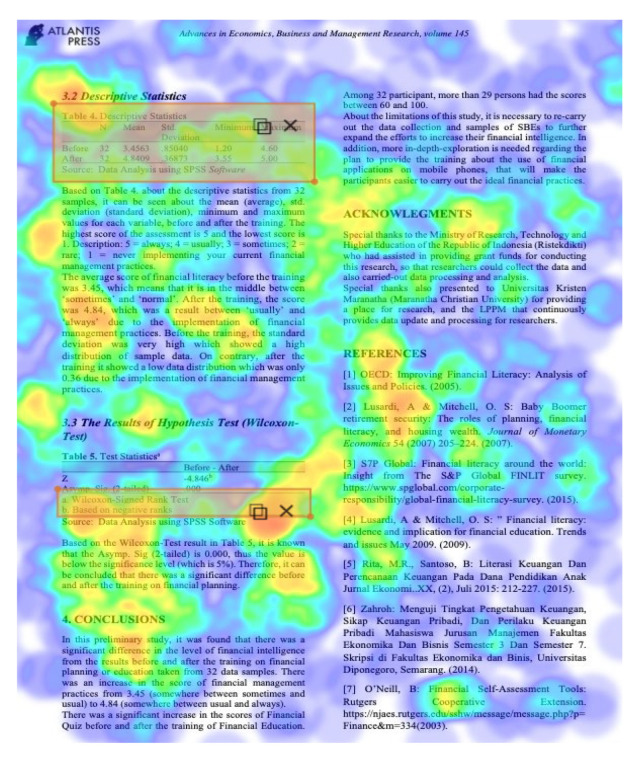
Heatmap of Image 6, a two-column proceeding paper layout.

To answer the first research question, we analyse several characteristics. The first one, as given in Images 1 and 4, is that learners are paying attention to something they recognize, such as the first language they are experts in. The FFD of Image 2 and Image 4 in Figure 4 also strengthens it. Both have two columns, but the learners pay more attention to Image 2, which is given in their mother tongue. If more than one language is used in a scientific paper, in the title or abstracts, it is suggested to place them in a side-by-side composition. The second important characteristic is that a two-column layout is preferable. Two columns give an extensive overview, and the learners might find more interesting information. In a two-column layout, the learners prefer a slimmer column. This fact can be deduced from the FFD in Image 3, Image 4, and Image 6. To strengthen these findings, we also present the heatmap of Image 6 in Figure 9, where it can be seen how the pre-test adult learners perceive the image on average. Most of them pay attention to the important information in the table.

**Figure 10:**
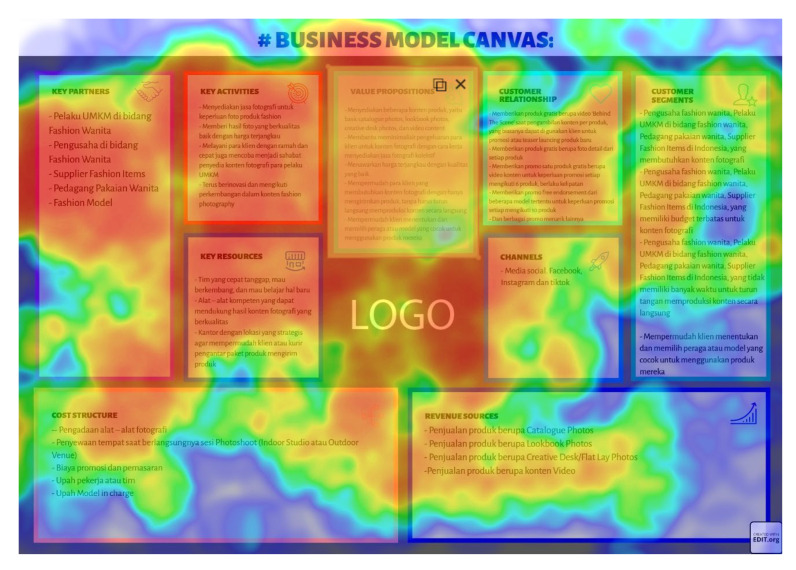
Heatmap of Image 8, a scientific poster with content in Indonesian Language.

Finally, regarding the characteristics of the poster, all learners prefer to see important information in the middle of the poster. This fact can be traced by analysing Figure 6 and Figure 7. The average distance to the AoIs for Image 8 is relatively close, quick to find, and lasts relatively long. The heatmap in Figure 10 also strengthens this fact.

### 5.2. RQ2: Which Characteristics Are Preferable for Specific Learner Groups?

From Figure 6, we can derive how adult and regular learners behave. The first-time leap during the pre-test of adult learners for scientific papers is generally higher than that of regular learners. This result suggests that adult learners concentrate better on details (small fonts) when they search for important information. However, from Figure 3, we can also deduce that lecturers need to instruct the students on specific details so they can find the important part of a piece of text.

In our observation, adult learners prefer textual representation. This group pay more attention to text compared with image. We also notice that AoIs, given a more comprehensive gap or space in the body text, are quickly read as an important part, even without enlarging the font size and making it bold. This consequence can be deduced from Image 1, where the TS duration is concise. We argue that these findings align with real-world conditions, where adult learners explicitly express the need for clear guidance and accurate exemplars to complete assignments more effectively within the limited time that they can dedicate alongside their other life responsibilities. Additionally, another study notes that adult students, lacking a sense of community, prefer assignments they can complete on their own schedule and value the learning materials provided [35]. Postgraduate adult learners require the ability to engage deeply with scientific content and maintain focused reading, as their education demands independent comprehension to build a foundation for conducting structured research and producing publications.

Our study shows that adult learners have specific characteristics regarding digital learning materials, especially scientific resources such as papers and posters. Compared to regular students, who are more image-based, adult learners show their ability to interpret textual content. Thus, we recommended that lecturers deliver digital content for adult learners with a reasonable amount of text, tables, and images. It is also recommended that adult learners be provided with several opportunities to express their learning outcomes through scientific outputs, such as posters, schematic diagrams, or scientific posters, to summarize their knowledge and demonstrate their understanding of the topic.

## 6. Conclusions

In this research, we have experimented with an eye-tracker tool to analyse how the layout of scientific papers and posters influences learners’ behaviours. Our findings show that the first language used in the textual document is the most important issue. This may suggest that adult learners engage more deeply when layout matches their reading habits. The other important aspect is the composition of text portions on a page. A side by side composition of essential information is preferable to a top-to-bottom arrangement. For a scientific poster, the learners tend to focus on the centre part, while left-to-right and top-down gaze movements are identified in the paper as this analysis is confirmed by the AoIs distances during the first, middle, and final time leap. Therefore, we recommend that lecturers design and present learning materials for adult learners in a textual format with a sufficient amount of information and/or by placing the important parts of posters in the center.

In subsequent research, we propose to continue the eye movement analysis with cognitive validation by longer fixation time. This objective could be accomplished by comparing it with descriptive surveys and grade analysis. We also plan to add more variations of papers and posters to validate the findings in future work.

The main limitation of this study lies in its heavy reliance on eye-tracking devices. Given that our experiments focus on a specialized profile of adult learners, the findings may only be generalizable to a subset of the digital immigrant population characterized by Latin-alphabet users (in this case, Indonesian speakers reading from left to right), the use of posters as canvases in the business domain (specifically employing the business model canvas), and the classification of the regular learner group as the comparison without differentiating tendencies based on their academic disciplines.

## Figures and Tables

**Table 1:** Image use in the experiments.

Image	Type and Features	Quantity and % of AoIs in the Image	Image Link with AoI (in the Red Box)
1.	Journal; Single column; Bilingual abstracts and keywords; Full first page	2 (abstracts middle (EN) and bottom (ID) AoIs); 7.08	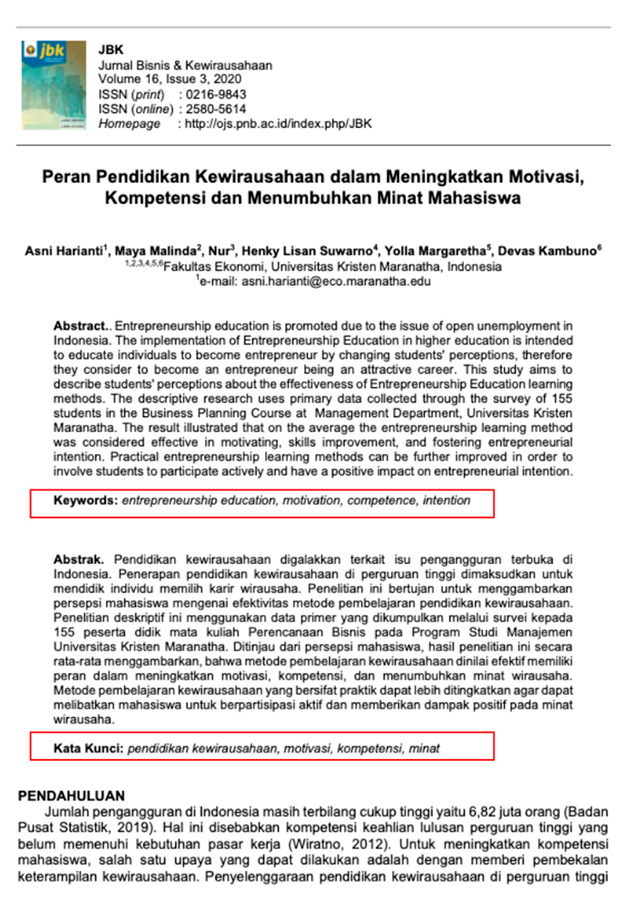
2.	Proceeding; Single column; Bilingual abstracts and keywords; Full first page	1; (abstract (ID)); 3.08	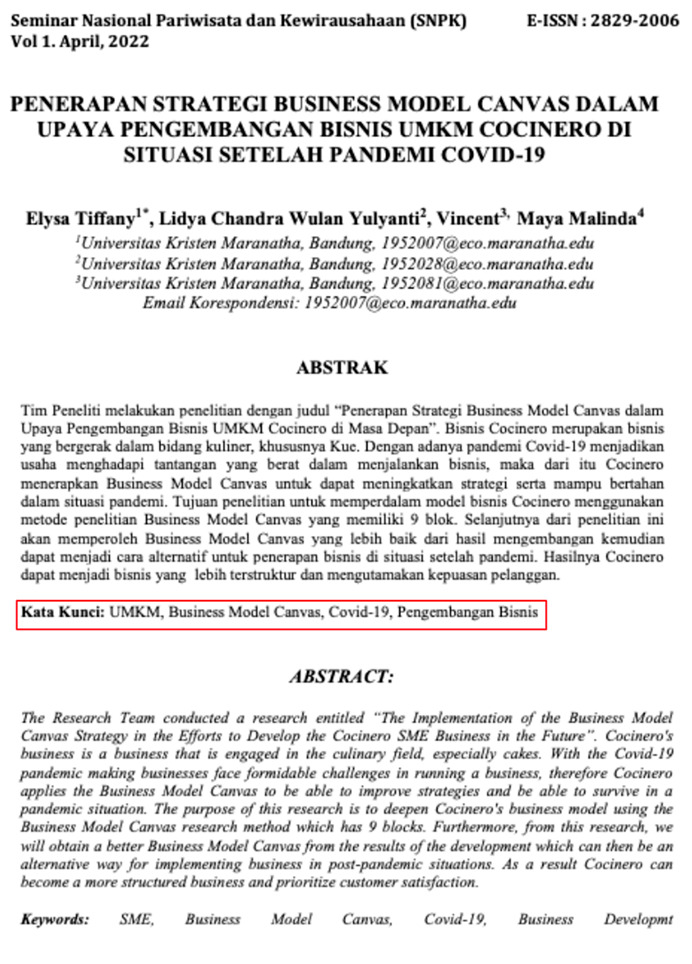
3.	Journal; Two columns; English abstract and keywords; Half page of the first page	1 (abstract (EN)); 4.75	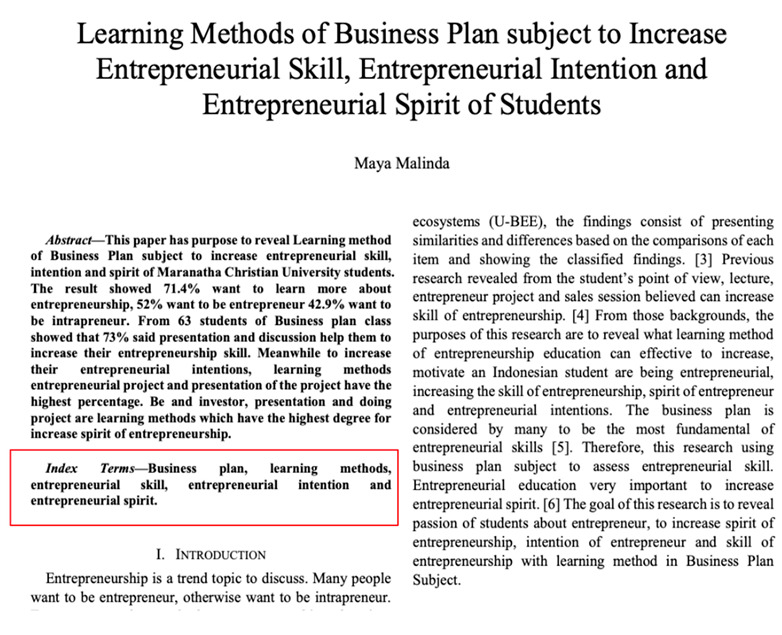
4.	Proceeding; Two columns; Bilingual abstracts and keywords; Full first page	2 (abstracts left (EN) and right (ID) AoIs close side by side); 5.32	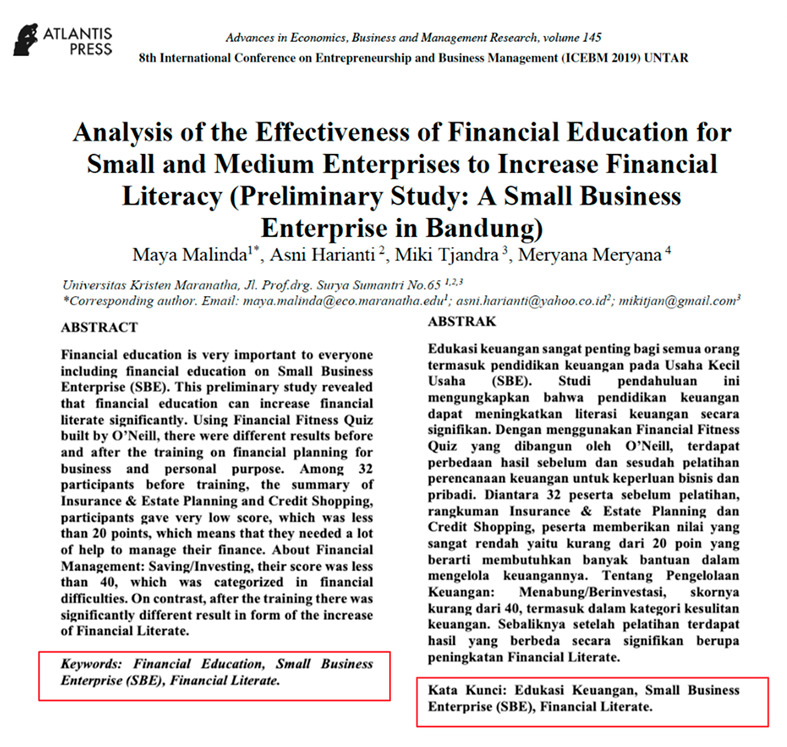
5.	Proceeding; Two columns; English abstract and keywords; Half page of the first page	1 (abstract (EN)); 2.99	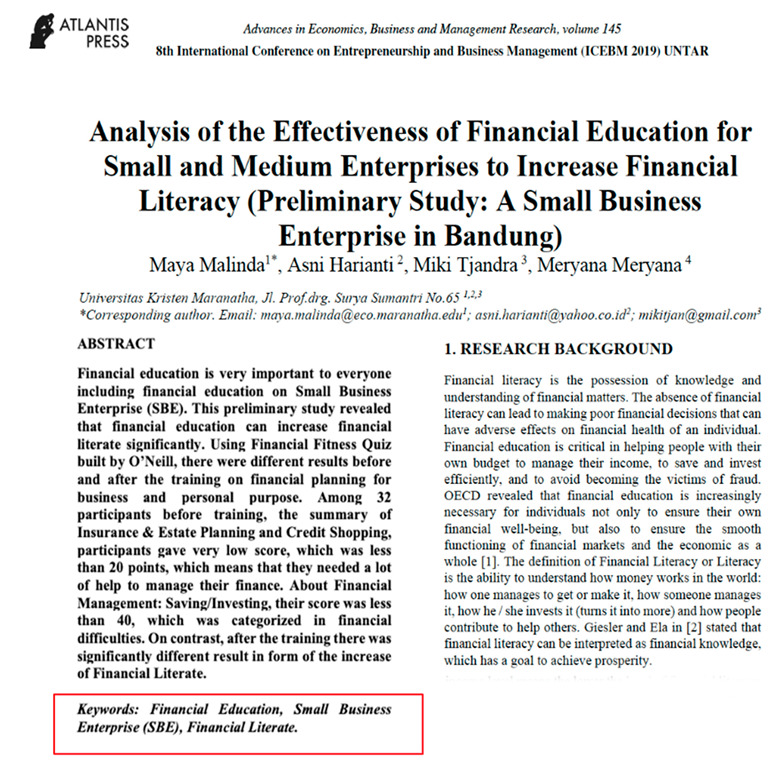
6.	Proceeding; Two columns; Full last page	2 (tables top-middle AoIs); 6.29	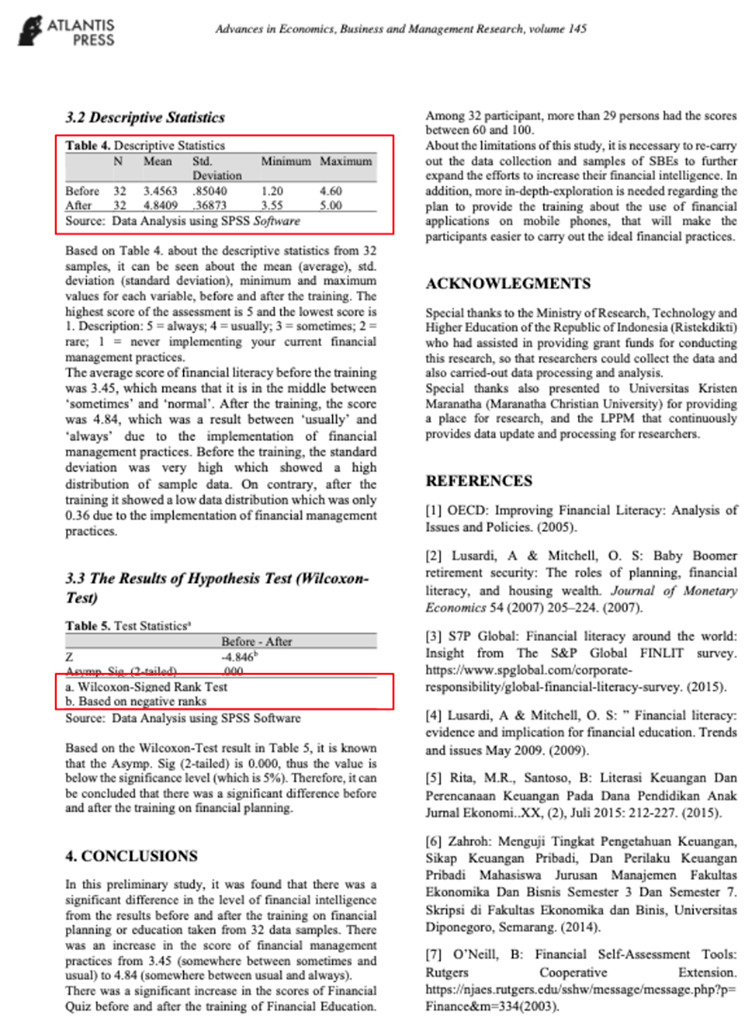
7.	Poster; Business model canvas template	1 (middle top row); 3.17	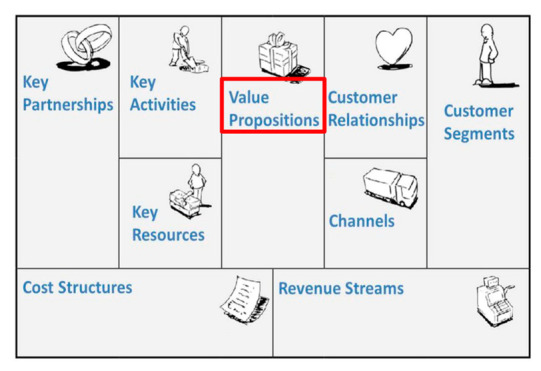
8.	Poster or canvas; Business model canvas with content in Indonesian Language	1 (middle top row); 7.03	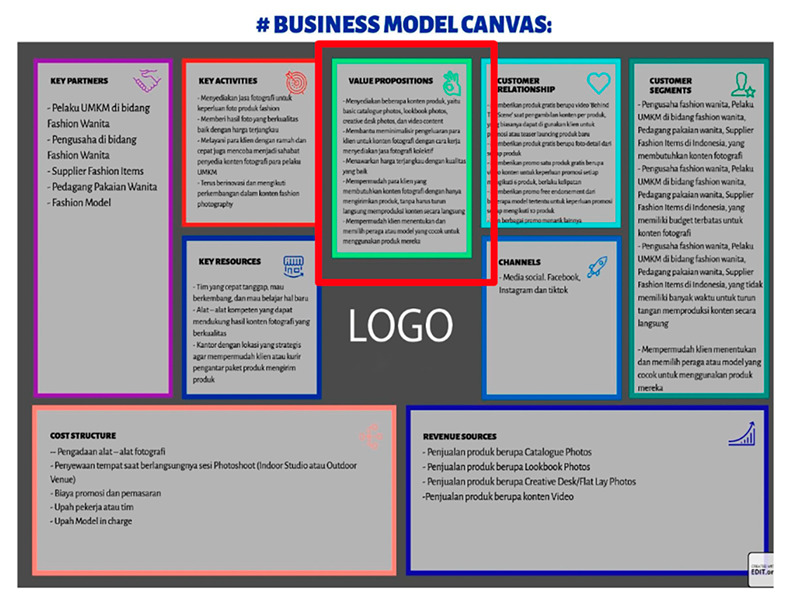

EN: English language. ID: Indonesian language.

**Table 2:** Significance test results of the experiments.

Image	Group	TTFF	FFD	TS
1	pre	7570.65	226.1	592.14
	post	**6128.38**	**250.76**	**1007.23**
2	pre	8661.47	253.8	8661.42
	post	**6665.35**	**306.71**	**6665.31**
3	pre	8441.71	235	8442.02
	post	**5681.84**	**278.79**	5682.18 *
4	pre	9550	183.25	9550.10
	post	**5742.83**	**265.33**	5743.37 *
5	pre	6696.55	220.45	6697.08
	post	7264.6 *	215 *	**7265.26**
6	pre	5604.70	257.75	5695.21
	post	**3369.04**	**278.6**	3369.38 *
7	pre	4720.63	291.47	4721.23
	post	**3879.56**	**300.24**	3880.18 *
8	pre	3136.30	245.13	3136.33
	post	7611.43 *	**249.96**	2784.05 *

* The opposite of the initial expectation of the pre- and post-tests.

## Data Availability

The original contributions presented in this study are included in the article. Further inquiries can be directed to the corresponding author.

## Abbreviations

The following abbreviations are used in this manuscript:

AoI	Area of Interest
TTFF	Time to First Fixation
FFD	First Fixation Duration
TS	Time Spent
